# Connected health services: Health professionals’ role as seen by parents of a child with inflammatory bowel disease

**DOI:** 10.1177/20552076241271772

**Published:** 2024-08-16

**Authors:** Aline Christen, Franzisca Domeisen Benedetti, Daniela Händler-Schuster

**Affiliations:** 1 Institute of Nursing School of Health Sciences, 30944 ZHAW Zurich University of Applied Sciences, Winterthur, Switzerland; 2 Institute of Nursing Science, Department of Nursing Science and Gerontology, UMIT TIROL Private University of Health Sciences and Health Technology, Hall in Tyrol, Austria; 3 School of Nursing, Midwifery and Health Practice, Te Herenga Waka, Victoria University of Wellington, Wellington, New Zealand; 4 247196 School of Public Health and Social Work, Faculty of Health at Queensland University of Technology, Brisbane, Australia

**Keywords:** Connected health, telemedicine, mobile application, inflammatory bowel disease, parents, child

## Abstract

**Objective:**

Connected health services will change the scope of health professionals’ roles. It is unclear how parents of a child with inflammatory bowel disease perceive the role of health professionals in relation to these services and what their experiences and needs are. The purpose of this study is to highlight parents’ experiences with this role. Furthermore, it aims to outline the fundamental needs that parents have regarding this role, in order to promote audience-specific access to these services and derive overarching action measures.

**Methods:**

Fourteen parents of children with inflammatory bowel disease from seven different clinics in Switzerland were recruited. Between August 2022 and February 2023, these parents were interviewed in semi-structured interviews. The interviews were analyzed using a structured qualitative content analysis.

**Results:**

Five main categories were identified, with few parents having prior experience with the role of health professionals in this area. Parents saw health professionals in the role of gatekeepers, transferers of knowledge and in a supporting function for these services. From the parents’ perspective, health professionals should recognize the limitations of these services and use them as a complement to standard treatment.

**Conclusion:**

The role of health professionals in relation to connected health services needs to be adapted from the parents’ perspective. To meet the needs of parents, health professionals must have access to these services. In addition to health professionals’ personal engagement with these services, institutional and policy changes, as well as research on role development from the perspective of other stakeholders are needed.

## Introduction

According to the United Nations and the World Health Organization, digitalization in the healthcare sector has greatly advanced in recent years.^1,2^ In Switzerland, this development is to be promoted and coordinated.^3^ Telemedicine such as health and medical applications (apps), among other services, exist to improve healthcare in the area of digitalization. According to Knöppler et al., these services are grouped as connected health and will not only have an enormous impact on the management of chronic diseases, but also on the role of health professionals.^4,5–7^ However, it is not obvious what experiences specific groups of patients and their relatives have already had with this role and what needs they bring with them.

Parents of children with inflammatory bowel disease (IBD) form a specific stakeholder group for connected health services. They are the primary caregivers for the disease management of their child, who is affected by a chronic illness, that presents physical and psychological challenges.^8,9^ Typical symptoms of IBD are abdominal pain, diarrhea, bloody stools, or loss of weight, which are often associated with lifelong therapy and a reduction in the overall quality of life.^10^ For this population, some connected health services, that can complement disease management, already exist.^11^ A wide range of telemedicine tools are available, which can improve access to care, increase quality of care, and reduce costs.^12,13^ In contrast, there are still fewer health and medical apps specifically developed for IBD than for other diseases and the majority are not designed for the pediatric setting. The available apps that are written in German contain information and serve as a means of exchange among those affected, but also between the affected persons and health professionals. Additionally, they can provide support in therapy management and thus improve education as well as the quality of life and treatment of patients.^14–18^ Despite these advantages, the utilization of such connected health services by health professionals and affected individuals varies across countries.^19–21^

According to Levesque et al., health professionals play an important role for parents when it comes to accessing connected health services. In the interdisciplinary care of children with IBD, it is mainly doctors and nurses, but also nutritionists and psychologists, who can influence the approachability, acceptability, availability, accommodation, affordability, and appropriateness of connected health services.^22^ In addition to facilitating access to these services, health professionals can use telemedicine and health and medical apps, to make their work more efficient and improve information exchange with patients.^23^ However, this situation is currently dominated by conflicting views and differing digital literacies among health professionals that influence parents’ access to these services.^24^ On the one hand, health professionals observe higher satisfaction and promotion of the patient’s self-competence through these services.^11,14^ On the other hand, health professionals are now suddenly confronted with the task of dealing with new technologies, some of which are perceived as inefficient.^25,26^ Although these obstacles exist, Seebach and Wasilewski state that the scope of health professionals’ work will have to adapt to the new realities in the future. Health professionals should provide individualized support to patients and their families who, by means of these services, would strengthen their self-determination.^27^ In these regards, initial descriptions of essential skills required for health professionals to effectively engage with connected health services are available.^28^

From a professional perspective, the direction in which the work of health professionals will move in the future is therefore predetermined.^27^ However, what this means specifically for the role of health professionals, in relation to connected health services, from the perspective of parents of a child with IBD, is not clear from the current literature.

The purpose of this study is to describe the experiences of affected parents with the role of health professionals in relation to connected health services and to highlight their needs for the development of this role. This will provide a general understanding of how health professionals currently support parents in this area. In addition, the needs of parents for the future role of health professionals will be identified, in order to be able to make needs-oriented recommendations for health professionals. With this knowledge, measures for action on the institutional and political level can be derived.

## Method

In this scarcely studied research area, a descriptive, qualitative design was used to be able to describe recommendations from the parents’ point of view.^29^ This study is based on the principles of interpretative description (ID). ID is a method, which provides a theoretical and flexible approach to analyze qualitative data. It focuses on developing a practical understanding of a phenomenon.^30^

### Setting and access to the research field

The target population for this study included parents of children with IBD up to 18 years of age. They are the main people responsible for the disease management of their child, who is confronted with lifelong health limitations and is dependent on permanent medical care.^31^ Parents living in Switzerland and speaking German or English were included, while parents of children diagnosed within the last three months were excluded, in order to protect families in this vulnerable phase.

Convenience sampling was used to obtain study participants. Seven children’s hospitals in German-speaking Switzerland with approximately 50,000 to 200,000 outpatients and 4000 to 8500 inpatients annually and the Swiss Crohn Colitis Association were contacted and provided with background information about the study.^32^ Various people from the medical, nursing, and administrative departments of these institutions informed affected parents verbally or in writing about participation in the study. Since few parents came forward through this recruitment step, promotional material for the study was also uploaded on two relevant Facebook pages. In addition, the method of snowball sampling was used, in which study participants informed other affected parents about participation. Interested individuals could contact the first author by mail or telephone. The first author provided the parents with comprehensive information about the study procedure and her professional background. The first author worked in one of the institutions in which parents were recruited for the study but had no personal contact with them prior to data collection. This was disclosed to all parents. Both written and oral informed consent was obtained from all participants who volunteered for the study.

### Data collection

Data collection took place between August 2022 and February 2023 at a location chosen by the participants. This was at their home, at a restaurant, or at the hospital. One person opted for online participation. The method of semi-structured individual interviews was selected to make the implicit individual knowledge of the parents visible and to structure the narrow topic area.^33^ The authors developed the interview guide based on the research questions, utilizing Helfferich's SPSS method for interview development, and incorporating Levesque et al.'s framework of access.^34^ The framework was used to ensure that all five dimensions of access to a service (approachability, acceptability, availability and accommodation, affordability and appropriateness) were covered in the interview guide.^22^ This interview guide was pilot-tested once by means of an interview that was ultimately integrated into the data analysis. A key question asked to all participants was: “What is your need to be supported by health professionals on the topic of connected health services?” All interviews were audiotaped and supplemented with interview notes, which contained general impressions and reflections of the interview from the interviewer`s perspective.

### Data analysis

The interview recordings were continuously transcribed verbatim and reviewed for the data analysis, whereby the interview guide was adapted in an iterative process.^29^ Subsequently, a structuring qualitative content analysis was conducted to ensure a rule-guided qualitative analysis.^35^ For this purpose, all the data material was initially open-coded and assigned to the deductively formed categories of *experiences* and *needs*. Only the themes relating to experiences and needs of health professionals’ role in association with connected health services were included. Expectations of digitalization itself or demands on health professionals that were not related to digitalization were excluded. Then, in an inductive step, according to the process of open coding, the categories were conceptualized, defined, and developed. For this purpose, the data material was initially sifted several times, which enabled peculiarities of interviews, but also similar concepts across interviews, to be crystallized. These similar concepts were grouped, compared with the interview notes and the dimensions of access from the framework of Levesque et al., and then finally structured in a coding framework.^22^ Through this process five main categories were developed, each of which was divided into the subcategories of experiences and needs. These subcategories contained a total of 23 further categories to which the open codes were assigned.

The analysis was completed with the first ten interviews and then tested on four more interviews by incorporating the newly generated open codes into the coding framework. MAXQDA 2022 software was used for the entire analysis process.^36^

### Quality criteria

The conduct of this study was based on the research ethics principles of the Helsinki Declaration and the Singapore Statement on Research Integrity and was reviewed by the Cantonal Ethics Committee Zurich.^37,38^ The entire research process was also guided by the quality criteria described by Elo et al. For this, the “Checklist for Researchers Attempting to Improve the Trustworthiness of a Content Analysis Study” was systematically followed in the planning, during, and after completion of the study.^39^

Specific to data analysis, the quality criteria of validity and reliability are additionally important, as recommended by Schreier.^35^ To promote reliability, the coding framework created was tested on the final four interviews. Data collection and analysis were conducted by the first author as part of her master's thesis, and validity was increased through regular meetings between the first and last author and an internal peer review process by reviewing, reflecting, and adjusting the research process. Differing views between the first and last author were resolved by iterative data analysis, in which the categories were formed in a multi-stage process. In addition, member checking was performed according to the recommendations of Birt et al. in which a summary of the results was sent to all study participants.^40^ Two responses received within 14 days were reviewed and included in the analysis.

## Results

Sixteen interested parents contacted the first author, although two mothers did not respond to the appointment requests in the course. Ultimately, 11 mothers and 3 fathers, whose children had been treated at seven different clinics, participated in single interviews. The parents interviewed ranged in age from 32 to 51 years, with an average of 44 years. The ages of their children ranged from three to 18 years, with an average of 13 years. The length of time since the children were diagnosed ranged from three months to seven years. Interviews lasted an average of 45 min. To preserve the anonymity of the study participants, only some of the demographic information described above is shown in Table 1.

**Table 1.** Demographics of study participants.

	Number (*n* = 14)
Parents’ gender	
Female	11
Male	3
Length of time since child was diagnosed	
<1 year	2
1–5 years	10
6–10 years	2
>10 years	—
Min./Max.	3 months/7 years

**Figure 1.**
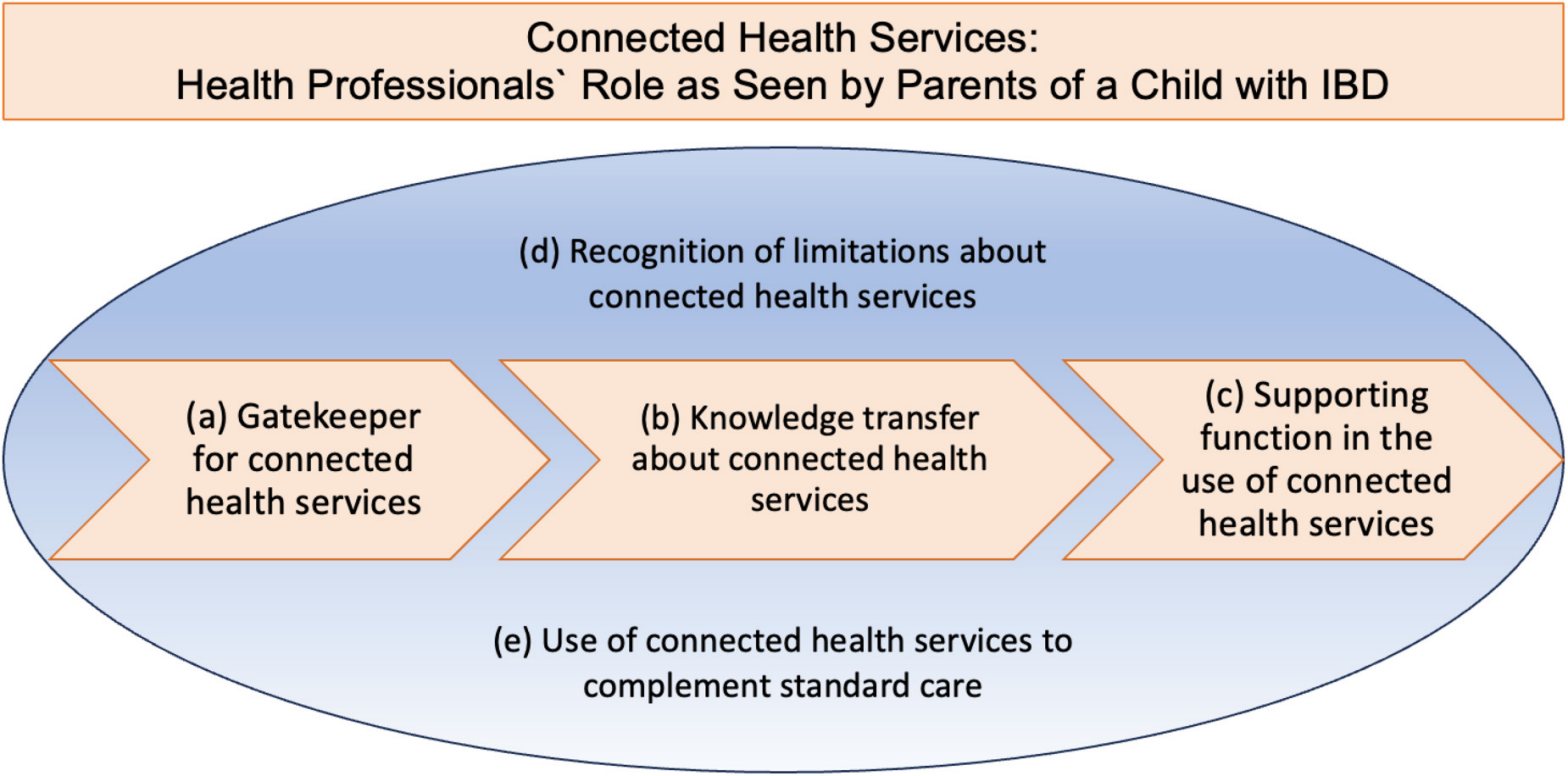
Connected health services: health professionals` role as seen by parents of a child with inflammatory bowel disease (IBD).

The results were grouped into five main categories representing parents' experiences with the role of health professionals in supporting them with connected health services and their resulting needs (Figure 1). Since few parents had previous experience with connected health services or the role of health professionals in this area, the scope of health professionals includes the role of gatekeeper, which is to enable parents to access connected health services (a). Health professionals should provide parents with knowledge about connected health services (b) and accompany them in the course of this topic (c). The basic principle is that health professionals should recognize the limitations of these services (d), but in return use them as a complement to standard care (e). These five main categories are described in detail below.

### Gatekeeper for connected health services

The majority of parents had not previously used telemedicine or health and medical apps. They had no experience with the specific role of health professionals in this area. As a result, some parents had not yet given much thought to their need for this role. Most parents, however, were aware that they had not yet had access to connected health services through health professionals and expressed outrage about this.

Actually, I never thought of the idea before and I'm actually a bit shocked that in hospital A there's just really nothing, (…) just really nothing available, just nothing, nothing at all. (Parent 7, female)

Another part of the parents had experiences with the role of health professionals in connected health services, in that the parents brought up the topic independently with the health professionals. In some cases, health professionals responded to this suggestion and, for example, agreed to a telemedicine consultation at the parents’ request. In other cases, health professionals expressed their opposition to such services.

And the doctor told me not to read too much about it (laughs). Yes, but what do I do, if we don't know it, I have to read about it, otherwise I don't know what it's about. And then I just looked for everything myself. (…) And also the apps. (Parent 13, female)

Few parents were given access to connected health services directly through health professionals. In this context, some parents were recommended to use a clinic internal app, which includes a digital medical record. In other cases, nutritionists offered their consultation via telemedicine. For the most part, parents were very positive about these options and used them regularly.

And of course we could have seen each other. But so far it has actually been enough with Zoom sessions. (Parent 11, male)

Although experiences of accessing connected health services varied widely, parents agreed that they should be informed about such services by health professionals.

I think it's simply a matter of bringing it to us parents in the first place. Yes, because if we don't get it, we can't use it (…) we just really need the information that it exists. (Parent 14, female)

From the parents` perspective health professionals should take on the role of gatekeeper to ensure that parents have access to connected health services.

### Knowledge transfer about connected health services

On the one hand, the few parents who were granted access to connected health services through the treating clinic were notified in writing via their appointment letter or through brochures about health and medical apps or telemedicine consultations. On the other hand, some also received verbal information from administrational staff.

Yeah, that app, they actually gave that to me at the first remicade therapy, when [daughter] left, she [administrative staff member] gave that to me in the reception office, actually just gave me all the paperwork. (Parent 8, female)

Parents hardly received any further information about connected health services directly from health professionals. In general, parents had the impression that the knowledge of health professionals on this topic was incomplete.

But I don't think that's a topic that's in the foreground for them, I have the feeling. (…) But basically the needs that one has or someone might have for internet access or internet information or apps. I don't think that's there yet, the intuition, or the knowledge. You really still have to build that up. (Parent 4, female)

Parents wanted health professionals to have knowledge about the services and to network on this topic. They saw the future role of health professionals as being responsible for producing written or digital documentation on the services and handing this out to parents. Most parents aimed for a short training sequence on these offerings, in which the benefits were communicated. Some also expected to receive information on measures to protect their data and few of them asked for support with the technical operation.

That would actually be the information that you would have to have from the hospital. From the doctor or from the nursing staff, who explains it. (…) This is the possibility and so you can use it and for that you can report there. (Parent 5, female)

From the parents' perspective, this training sequence could also be supplemented with video material or information sessions about connected health services. As for the timing of this training, parents were not in agreement. Some said that they would have been overwhelmed with information about these services shortly after receiving the diagnosis. Others said that this training would have been important, especially at the beginning.

### Supporting function in the use of connected health services

Parents were hardly accompanied by health professionals on the subject of connected health services. On the contrary, health professionals tended to make negative comments about the services when parents had accessed information via an app with a digital medical record, for example.

And then she came in and she said that the results were available. I said, yes, I saw them. And then she said, she didn't like it. She doesn't think it's good at all! That we parents then see some of the results before they see them. Yes. That was such a remark. (Parent 5, female)

A few parents said that accompaniment in the course was not important for them. They felt that a one-time provision of information was sufficient. For most parents, however, the future remit of health professionals included professional support in the use of connected health services. Parents indicated that regular exchange and obtaining feedback on the topic could motivate them to use the offerings.

At the second, third, when you come back, maybe ask if it's of any use. Maybe such a thought-provoking or such a nudge would perhaps also be good. Also important to stick with it. (Parent 4, female)

Parents wanted the use of connected health services to be evaluated jointly by health professionals and parents. In addition, health professionals should be available to parents in an advisory capacity if parents have further questions.

Contact and exchange person. Or even if I bring up something, the doctor doesn't say, yes, I've never heard of that, but, ah, yes, that's right, I've read something about that in this app. (Parent 12, female)

From the parents' perspective, connected health services should be integrated into the existing service of health professionals, making them a permanent part of the treatment.

### Recognition of limitations about connected health services

In addition to the potential of connected health services, parents also identified the disadvantages of these options, which health professionals should be aware of and respect. Parents, who were very satisfied with their child's treatment, who had a clear contact person, or whose child had been diagnosed for a longer time were less likely to express a need for connected health services.

But, yes, it is somehow, he is already so long in remission and then somehow you just live and do not think so much about the disease (…) I would be aware of this app, when it goes into the direction of a flare again, when one then needs more help again. But just, if he is so, if he is almost like healthy (laughs), so to speak, then yes, I think, then I would need it less now. (Parent 14, female)

In addition, parents considered the physical examination of their child very important and the physical exchange with health professionals supported the trust relationship.

A little bit of trust, perhaps. (…) But I still prefer it when it is personal. I'm one of those who have a bit of trouble with telemedicine. (Parent 9, female)

Parents said that health professionals should be sensitive when using connected health services and need to weigh in with them when they are appropriate.

So, the further procedure, change of therapy, may also be discussed in this way. Yes. But just the first conversation, when you really get a diagnosis from a healthy, from a healthy child, that you really sit together. (Parent 6, female)

In principle, these services should provide relief for parents. Many parents reported that they did not have time for additional effort regarding connected health services. They pointed out that the financing of these services needs to be clarified. Additional costs, which would be incurred by these, would be an obstacle for many parents in using them. Because of these disadvantages, parents appealed for individualized and voluntary treatment with connected health services.

### Use of connected health services to complement standard care

From the parents' point of view, the attitude of health professionals should change so that they can use connected health services as a supplement and thus close gaps in conventional treatment. A large proportion of parents said that the first period after diagnosis or the acute phases of the disease was particularly challenging. They felt overwhelmed, insufficiently informed, and would have liked more support.

Yes, so actually, so to speak after the diagnosis, so to speak the therapy support has been quite missing. (…) And of course you are like, so to speak, you have ten questions a day, because you are doing this for the first time and the child doesn't know what is right and wrong and we don't know whether this is good or bad, how the child reacts. And, and, there of course I would have liked more guidance. (Parent 11, male)

Parents saw connected health services as a way for health professionals to help them with these challenges. Telemedicine such as health and medical apps could be used to meet their need for information.

I just want as much information as possible. And I therefore take it where I get it! (Parent 5, female)

According to the parents, communication and exchange with health professionals could be promoted by telemedicine. Especially in the case of urgent or short requests, parents would like to make use of this possibility. Many parents could also imagine partially replacing consultations in the hospital with telemedicine consultations. This would help both parents and health professionals to organize appointments.

But I have now often experienced, now we are in the children's hospital, where I thought, ok, that could have been done now really from home, because it is a big effort for us. I always need a babysitter for them. Then the distance, it's also 40 minutes, so it's a big effort (…) you can also, I think by, yes, telemedicine consultation. (Parent 3, female)

The parents spoke of various health professionals who should adapt their scope of duties through these services, namely doctors, nurses, nutritionists, and psychologists. The only thing parents agreed on was that doctors and nurses should not only offer telemedicine consultations, but that physical consultations are also necessary. Otherwise, views differed on the various roles of different professions involved in this field. However, parents shared the view that the role of health professionals needs to be adapted.

So, from that point of view, the most the hospital can do is simply grab digitalization by the horns, (…) and inform itself there and approach it more actively. (Parent 11, male)

Parents recognized that digitalization had not yet advanced in their child's treatment and called for a change in the role of health professionals so they could take advantage of connected health services for themselves and their family.

## Discussion

This study explored the experiences of parents of children with IBD with the role of health professionals in connected health services and which needs parents bring to health professionals on this topic. The results of this study illustrate that few parents have experience with connected health services or the role of health professionals in this field. This is conditionally consistent with other studies in this area.^19–21^ The present study was conducted in a German-speaking country and with a specific population of parents of pediatric patients. It is assumed that health professionals are reluctant to use telemedicine with this population and that the limited availability of health and medical apps has an influence on their use. Despite the lack of experience, parents already have a clear idea of how such opportunities could be used and how they could be supported by health professionals. This may be due to the fact that parents are familiar with comparable digital services from their everyday lives and transfer this knowledge to their child's treatment.^27^ Consequently, parents feel the need to adapt the tasks of health professionals, who are seen in the role of gatekeepers, so that parents have access to connected health services in the first place.^22^ From the parents' point of view, health professionals should impart knowledge about connected health services, support them with it, recognize the limitations of these possibilities, and use such services as a supplement to standard treatment.

As reported in the results of this study, adopting the role of health professionals in this area can improve the approachability and acceptability of connected health services for parents, as also described by Levesque et al.^22^ As far as can be assessed, this was the first study to pay attention to this specific topic. Other studies in this field focus on the attitudes and skills of health professionals in the area of digitalization. Although the results and recommendations of these studies are along the same lines, they shed light on the topic at a higher level of abstraction rather than in concrete terms.^5,24,25,28^

It is interesting to note that parents' expectations are mainly limited to the use of telemedicine consultations and health and medical apps that only include information. The parents surveyed did not speak of wishes for more advanced connected health services, as is the case for diabetes care, for example. In this field, digitalization is used to process and distribute information, and to make and control decisions.^41^ This may be due to the fact that parents have hardly dealt with this topic yet. Additionally, IBD is a complex and less common disease.^9^ This makes the use of such services very challenging from an economic and professional point of view and thus limits availability, affordability, and appropriateness.^19,22^ Furthermore, the results suggest that not only health professionals, but also parents are characterized by conflicting views and differing digital literacies. In particular, parents whose child was in a stable situation and who had a clear medical contact person expressed a reduced need for connected health services.^24^

To enable health professionals to address parental needs and adapt their role in the area of digitalization, they also need access to connected health services, which have only been available to date in rudimentary form. At the micro level, this includes the personal interest of each individual health professional in approaching this topic and informing themselves.^26^ Even if the involvement of professionals and the scientific evidence in the development of the current offers are partly incomplete, there are already several telemedicine tools, to which parents can be given access and for which risk assessments are made.^13,42^ In addition, there are some apps that can be recommended to parents that are more likely to be classified as health apps and not as medical devices.^15–18^ Such apps are recommended, for example, by self-help groups or on relevant websites, partially provided by pharmaceutical companies.^15,16^ These health apps have the primary goal of promoting health and do not directly support parents in disease management, which means they are associated with a smaller risk.^4^ In order to meet the needs of parents, it is the task of health professionals to consider such low-threshold services, taking into account the individuality of each family.^43^

According to Konttila et al., at the meso level, inter-organizational guidelines for health professionals need to be established, regulating the handling and new tasks of health professionals with connected health services.^24^ Institutions must recognize and guide the opportunities and risks on both sides for the families involved, but also for their employees. In addition, it is their responsibility to adhere to overarching recommendations and to provide the necessary digital equipment.^44,45^ This means that further development of the role is not only geared to the needs of parents but also makes sense from a professional and economic point of view.

In order for a change in the scope of health professionals to take place in line with the recommendations of parents, political preconditions must be met at the macro level, for which strategies have already been developed in Switzerland.^3,45^ In addition to the regulation of connected health services, which are currently more driven by supply and consumption, there is a need for more incentives for health professionals who can create access to such offerings for parents.^3^ Health professionals demand high-quality products that can also be financially settled by them.^46^ These services must be developed in such a way that they become more efficient, while currently they are mostly connected with an additional time expenditure for health professionals.^25,26^ Furthermore, health professionals must be offered the opportunity to acquire skills in dealing with connected health services. It is therefore necessary to include this topic in the basic training of health professionals and create dedicated further training courses.^12^ Last but not least, connected health is an interdisciplinary issue. In the present study, parents do not agree on the different disciplines to which their recommendations should apply. According to Zakaria et al., interdisciplinary dialogue, clarification of responsibilities, and the creation of new fields of work are necessary in order to meet the demands of parents.^47^

## Strengths and weaknesses of the study

Even though the inclusion of 14 parents is a limited number, participants from seven different hospitals in German-speaking Switzerland took part to this study, ensuring a broad insight into the experiences and needs of parents. Since convenience sampling was conducted, selection bias is to be expected. It is likely that parents who have a greater interest in connected health services were the primary respondents, which may have been reflected in the results. However, the education level of the parents and the medical history and treatment of the children were not recorded and analyzed in this study. In addition, it was not possible to include patients' perspective, which needs to be examined in further research.

Given the under-researched nature of this topic, a validated questionnaire was not available for data collection. Therefore, the authors developed a questionnaire following a structured procedure and conducted a pilot test prior to data collection.^34^ The interviews and analysis of this study were led by the first author, but they were regularly discussed with the last author and in a peer setting.^35,39^ Furthermore, the results were validated using member checking.^40^

In general, digitalization is an interdisciplinary topic and it would have been desirable to include various health professions in this work, since all authors of this study are members of social and nursing sciences.^47^ Additionally, IBD is a specific disease, which means that the results cannot be used to draw conclusions about the experiences and needs of people affected by other diseases or the perspective of health professionals. Nevertheless, this study has succeeded in providing a deep insight into this rarely studied topic.

## Conclusions

Parents of children with IBD have little experience with the role of health professionals in connected health services. As parents recognize the potential of these services, from their perspective, the role of health professionals in this topic needs to be adapted. The following implications for practice and research can be derived:

- Health professionals should use existing connected health services to complement standard care for children with IBD. For this to succeed, health professionals have the task of providing parents with access to these services. They should impart knowledge to the parents on this topic, support them in this, but also know the limitations of these offers.
- Health professionals themselves need access to connected health services. On the one hand, this requires an expansion of evidence-based offerings and the personal involvement of health professionals with this topic. On the other hand, institutional and political adjustments are needed so that health professionals can acquire appropriate competencies in dealing with connected health services, services can be regulated, and newly arising tasks can be distributed in a meaningful way.
- This study explored the experiences and needs of parents of a child with IBD and thus a specific population. In order for the role of health professionals to evolve in relation to connected health services, additional research is needed not only on influencing factors but also on role development from the perspective of other populations and stakeholders.

These arrangements will succeed in meeting the demands of parents while incorporating the needs of health professionals as well as the professional and economic demands of this challenge in changing the role.

## Supplemental Material

sj-pdf-1-dhj-10.1177_20552076241271772 - Supplemental material for Connected health services: Health professionals’ role as seen by parents of a child with inflammatory bowel disease Supplemental material, sj-pdf-1-dhj-10.1177_20552076241271772 for Connected health services: Health professionals’ role as seen by parents of a child with inflammatory bowel disease by Aline Christen, Franzisca Domeisen Benedetti and Daniela Händler-Schuster in DIGITAL HEALTH

sj-pdf-2-dhj-10.1177_20552076241271772 - Supplemental material for Connected health services: Health professionals’ role as seen by parents of a child with inflammatory bowel disease Supplemental material, sj-pdf-2-dhj-10.1177_20552076241271772 for Connected health services: Health professionals’ role as seen by parents of a child with inflammatory bowel disease by Aline Christen, Franzisca Domeisen Benedetti and Daniela Händler-Schuster in DIGITAL HEALTH
