# Supplementary material for: Connected health services: Health professionals’ role as seen by parents of a child with inflammatory bowel disease
Source: Digit Health. 2024 Aug 16;10:20552076241271772. doi: 10.1177/20552076241271772 (PMC11329971; doi:10.1177/20552076241271772)
Supplement: sj-pdf-1-dhj-10.1177_20552076241271772 - Supplemental material for Connected health services: Health professionals’ role as seen by parents of a child with inflammatory bowel disease [file sj-pdf-1-dhj-10.1177_20552076241271772.pdf]

Questionnaire

Connected Health Services: Health Professionals` Role as Seen by Parents of a Child with Inflammatory Bowel Disease

Interviewframe			
Date		Location	
Name interviewer		Interview number	
Start time		End Time	

Preparation:

- The interviewer introduces him/herself and the objectives of the interview.
- This is followed by a brief description of connected health services.

**1 Entry phase: The parent`s experiences**	
1.1	I am interested in how you use digital services in relation to your child's illness (apps, telemedicine):<br><br>- What is your need to use such services?
1.2	How did you become aware of these services (apps, telemedicine)?<br><br>- How did discussions about these services with health professionals look like?<br>- How do you rate the knowledge of health professionals on this topic?<br>- Which health professionals have you spoken to about this topic?
1.3	I am interested in how you were supported by health professionals in these services:<br><br>- What did health professionals do to make you aware of these services?<br>- Can you tell me about any positive experiences?<br>- Can you tell me about any negative experiences?
**2 Main Phase: The parents`s needs**	
2.1	What is your need to be supported by health professionals on the topic of connected health?<br><br>- How does that look like in concrete terms?<br>- What would be the recommendations for professionals?<br>- What would be the facilitating factors?<br>- What would be the hindering factors?<br>- How much time would be needed for this endeavor?<br>- Which professions would this affect?<br>- What could professionals do to increase your use of digital services?
**3 Final Phase: Open topics**	
3.1	Is there anything else you would like to say on this topic that has not yet been discussed?

**Socio-demografic data**	
Sex	
Age	
Age of the affected child	
Length of time, since the child was diagnosed	
Name of the clinic	

**Checklist**	
	Informed consent signed
	Asked whether the person may be contacted again
	Questions of the participant answered
	Made aware of snowballsampling
	Thanked
